# Transient multifocal genomic crisis creating chromothriptic and non-chromothriptic rearrangements in prezygotic testicular germ cells

**DOI:** 10.1186/s12920-019-0526-3

**Published:** 2019-05-28

**Authors:** Atsushi Hattori, Kohji Okamura, Yumiko Terada, Rika Tanaka, Yuko Katoh-Fukui, Yoichi Matsubara, Keiko Matsubara, Masayo Kagami, Reiko Horikawa, Maki Fukami

**Affiliations:** 1 Department of Molecular Endocrinology, National Research Institute for Child Health and Development, Tokyo, 157-8535 Japan; 2 Department of Advanced Pediatric Medicine, Tohoku University School of Medicine, Tokyo, 157-8535 Japan; 3 Department of Systems BioMedicine, National Research Institute for Child Health and Development, Tokyo, 157-8535 Japan; 4 Division of Endocrinology and Metabolism, National Medical Center for Children and Mothers, Tokyo, 157-8535 Japan; 5 Department of Neonatology, Aiiku Hospital, Tokyo, 105-8321 Japan; 6 National Research Institute for Child Health and Development, Tokyo, 157-8535 Japan

**Keywords:** Chromoanagenesis, Chromosomal instability, Chromothripsis, Comparative genomic hybridization, Complex genomic rearrangement, Copy number variation, Genomic imprinting, Genotyping, Haplotype, Whole genome sequencing

## Abstract

**Background:**

The co-occurrence of multiple de novo copy number variations (CNVs) is a rare phenomenon in the human genome. Recently, an “organismal CNV mutator phenotype” has been reported to result in transient genomic instability introducing multiple de novo CNVs in primary oocytes and early-stage zygotes. These findings opened a new area of human genome research.

**Methods:**

We performed genome-wide copy number analysis for ~ 2100 individuals with various congenital defects. Furthermore, extensive molecular analyses, including synthetic long-read whole-genome sequencing and haplotype-phasing, were carried out for an individual with multiple de novo CNVs.

**Results:**

A boy was found to have de novo rearrangements on five chromosomes. The rearrangements comprised simple duplication and inversion as well as chaotic changes, all of which affected paternally derived chromosomes. Postzygotic genomic instability was ruled out. The duplicated regions on 6q and 13q contained both diallelic and triallelic loci, indicating that the genomic rearrangements were initially created during premeiotic mitosis and subsequently modified by physiological cross-over during meiosis I. Breakpoints of the rearrangements were indicative of non-homologous end joining, replication-based errors, and/or chromothripsis. The mutagenic event was independent of specific local DNA motifs or de novo point mutations, but may be driven by spermatogenesis-specific factors.

**Conclusions:**

These results indicate that during spermatogenesis, a transient multifocal genomic crisis can introduce several chromothriptic and non-chromothriptic changes into the genome. These findings broaden the concept of the “organismal CNV mutator phenotype”. This study provides insights into mechanisms for altering the global chromosomal architecture of human embryos.

**Electronic supplementary material:**

The online version of this article (10.1186/s12920-019-0526-3) contains supplementary material, which is available to authorized users.

## Background

During gametogenesis, human chromosomes occasionally acquire de novo structural alterations. Non-allelic homologous recombination (NAHR), non-homologous end joining (NHEJ), and replication-based errors are known to underlie de novo rearrangements [1]. In addition, catastrophic cellular events designated as chromothripsis and chromoanasynthesis have recently been implicated in complex rearrangements involving one or a few chromosomes [1–5]. Chromothripsis likely occurs during spermatogenesis and postzygotic mitosis, but not during oogenesis [1, 6].

The co-occurrence of multiple de novo intrachromosomal rearrangements is an uncommon phenomenon that cannot be explained by the aforementioned mechanisms. In 2017, Liu et al. analyzed copy number variations (CNVs) in ~ 60,000 individuals and identified five cases with 5–10 de novo CNVs [7]. Interestingly, most of these multiple de novo CNVs (mdnCNVs) were large non-recurrent duplications, a rare type of human chromosomal rearrangement [5, 7]. These mdnCNVs predominantly affected maternally derived chromosomes and showed no signs of somatic mosaicism. Liu et al. proposed that during oogenesis and early embryogenesis, an “organismal CNV mutator phenotype” could create mdnCNVs [7]. This mutator phenotype appears to be driven by an unknown factor that is activated in primary oocytes and subsequently lost or silenced in zygotes before the 4- or 8-cell stage. Notably, one of the five cases (mCNV7) carried CNVs only on the paternally derived chromosomes, raising the possibility of etiological heterogeneity among mdnCNVs. These findings opened a new area of human genome research.

Here, we report a boy with mdnCNVs. The genomic structure of this case showed not only some similarities but also striking differences to those of the previously reported mdnCNV cases, suggesting a unique mutagenic event exclusively operating during spermatogenesis.

## Methods

### Primer sequences

Sequence information of primers used in this study is available upon request.

### Genome-wide copy number analysis for ~ 2100 individuals

We analyzed CNVs in the genome of ~ 2100 individuals with various types of developmental defects. Salient clinical features of these individuals were congenital malformations, growth failure, mental retardation, and/or disorders of sex development. Most participants were children of Japanese origin.

Genomic DNA samples were obtained from peripheral leukocytes or buccal swabs of the participants and subjected to array-based comparative genomic hybridization (aCGH) using catalog microarrays (SurePrint G3 human CGH; Agilent Technologies, Santa Clara, CA, USA). The experiments were carried out according to the manufacturer’s instructions. The results were analyzed using the Genomic Workbench 7.0 (Agilent Technologies). We searched for cases carrying multiple large (≥ 0.1 Mb) CNVs. Known recurrent CNVs in the Database of Genomic Variants [8] were excluded from further analyses. We also analyzed DNA samples obtained from the patients’ parents to exclude inherited CNVs.

### Clinical and cytogenetic analyses of a patient with mdnCNVs

A patient with mdnCNVs was subjected to clinical analysis. Furthermore, the patient underwent conventional cytogenetic analyses. G-banding analysis was performed using lymphocyte metaphase spreads of 20 cells (SRL Inc., Tokyo, Japan). In addition, we performed multicolor fluorescent in situ hybridization to determine the presence or absence of interchromosomal translocations. The experiments were carried out according to a standard protocol (LSI Medience Corporation, Tokyo, Japan).

### DNA methylation analysis

We analyzed DNA methylation indexes of seven CpG sites in the differentially methylated region at 6q24.2 for the patient with mdnCNVs*.* These CpG sites are located within a differentially methylated region adjacent to *PLAGL1*, an imprinted gene involved in glucose homeostasis [9, 10]. Usually, these CpG sites are methylated when transmitted from the mother and remain unmethylated when transmitted from the father [11]. Hypomethylation of these CpG sites is known to result in transient neonatal diabetes mellitus (TNDM) via *PLAGL1* overexpression. Pyrosequencing was performed as described previously [12]. In brief, a genomic DNA sample obtained from the patient’s leukocytes was treated with bisulfite using the EZ DNA Methylation-Gold Kit (Zymo Research, Irvine, CA, USA). The genomic region encompassing seven CpG sites was PCR-amplified using 5′-biotinylated and unlabeled primers. The PCR products were hybridized to a sequencing primer and loaded on the PyroMark Q24 sequencer (QIAGEN, Hilden, Germany). Methylation indexes were calculated using the PyroMark CpG Software (QIAGEN). The results of the patient were compared with the reference data obtained from 49 unaffected Japanese individuals [11].

### Further copy number analysis of the patient

To examine the presence or absence of somatic mosaicism and postnatal genomic instability of the patient, we performed further copy number analysis. Genomic DNA samples were obtained from peripheral leukocytes and oral swabs of the patient at 2 weeks and at 8 months of age, and subjected to aCGH. We calculated the average log ratios of signal intensity for probes located within the CNVs. Theoretically, the average log ratios for probes in non-mosaic heterozygous deletions and duplications are − 1.0 and + 0.58, respectively. Therefore, the average log ratios significantly higher than − 1.0 (for probes in deletions) or lower than + 0.58 (for probes in duplications) were considered as signs of mosaicism.

### Microsatellite analysis

Microsatellite analysis was performed for six loci in the duplicated regions on 6q and 13q. The methods were described previously [13]. In brief, genomic DNA samples of the patient and his parents were PCR-amplified using 5′-FAM-labeled and unlabeled primers. The PCR products were loaded on the Applied Biosystems 3130xl Genetic Analyzer (Thermo Fisher Scientific, Waltham, MA, USA). The results were analyzed using GeneMapper Software 3.7 (Thermo Fisher Scientific). Copy numbers of paternally and maternally derived alleles in the patient were estimated by calculating the areas under the curve of PCR products.

### Short-read paired-end whole genome sequencing

Genomic DNA samples obtained from peripheral leukocytes of the patient and his mother were subjected to paired-end whole genome sequencing. Genomic libraries were prepared using the TruSeq DNA PCR-Free Library Preparation Kit (Illumina, San Diego, CA, USA) and loaded on a HiSeqX sequencer (Illumina). Sequence data were obtained as 150-bp paired-end reads (GENEWIZ, South Plainfield, NJ, USA). Base calling and quality scoring were performed using Real Time Analysis 2.7.7 (Illumina). The base call files were converted to FASTQ files using the bcl2fastq Conversion Software 2.17 (Illumina). We removed the library adaptor sequences using cutadapt 1.14 [14]. We also trimmed low quality bases (quality scores of less than 16) at both ends and removed short reads of less than 72-bp. Sequence reads were mapped against the human genome reference (hg19/GRCh37) using Burrows-Wheeler Aligner (BWA) 0.7.15 [15]. We used Picard 2.8.1 (Broad Institute, Cambridge, MA, USA) to remove duplicate reads and to verify mate-pairs. Local realignment and base quality score recalibration were performed with the Genome Analysis Toolkit 3.5 (Broad Institute). BAM file data were visualized on the Integrative Genomic Viewer (IGV; Broad Institute).

### Synthetic long-read whole genome sequencing and haplotype-phasing

Haplotype-phasing of the patient and his father were performed through synthetic long-read whole genome sequencing (Macrogen, Seoul, South Korea). Genomic DNA samples were extracted from leukocytes. Sequencing libraries were prepared using the 10x Genomics Chromium System (10x Genomics, Pleasanton, CA, USA) and loaded on the HiSeqX sequencer (Illumina). Sequence data were obtained as 150-bp paired-end reads. Base calling and quality scoring were performed using Real Time Analysis 2 (Illumina). The base call files were converted to FASTQ files using the Long Ranger 2.1.3 (10x Genomics). Then, Long Ranger 2.1.5 (10x Genomics) was used to align sequence reads and to call single nucleotide variants (SNVs), indels, and structural variants. The output data were visualized on the Loupe 2.1.2 (10x Genomics). We genotyped SNVs of the patient and his father and constructed haplotype-phase blocks. In this analysis, we focused primarily on genomic regions flanking the breakpoints.

### Genotyping of SNVs in the duplicated regions

We referred to the whole genome sequencing data of the patient and his parents, to determine the number of alleles involved in the patient’s duplications. To this end, we selected SNVs in the duplicated regions for which the father and the mother were heterozygous and homozygous, respectively, and the patient was heterozygous with the minor allele accounting for 26–40% of the total sequence reads. We assessed the genotype of the patient as diallelic and triallelic when the reads of the father-specific allele accounted for approximately 2/3 and 1/3 of the total reads, respectively.

### Breakpoint characterization

To determine the precise genomic position of each breakpoint, we visualized the short-read whole genome sequencing data on IGV and searched for discordant paired-end reads. We referred to the results of G-banding analysis and aCGH to decide the candidate regions. We also analyzed the data of synthetic long-read whole genome sequencing on Loupe 2.1.2 (10x Genomics) to detect breakpoints that were missed by the G-banding analysis and aCGH.

The junction structures were determined by direct sequencing of the PCR-amplified DNA fragments harboring the fusion points. To this end, we performed PCR reactions using serial primers flanking the breakpoints. The genomic position of each breakpoint was decided against the human genome reference (hg19/GRCh37).

### In silico analyses for the breakpoint-flanking regions

In silico analyses were carried out for genomic regions at the proximal and distal sides of each breakpoint. First, we searched for microhomologies and microhomeologies at the breakpoints. Microhomology was defined as 100% identical DNA sequences less than 70 nucleotides, while microhomeology was defined as five or more nucleotides with 70% or more homology, without > 2 consecutive nucleotide gaps. We analyzed 20-bp sequences at the proximal and distal sides of each breakpoint using LALIGN (Swiss Institute of Bioinformatics, Lausanne, Switzerland) [16]. We set the opening gap penalty to 0 to detect all candidate sequences. The detected sequences were evaluated manually. Second, we examined the presence or absence of repetitive sequences within 2-kb regions at the proximal and distal sides of each breakpoint. Long interspersed nuclear elements (LINEs), short interspersed nuclear elements (SINEs), and human endogenous retroviruses (HERVs), were searched using RepeatMasker [17]. Third, we checked whether the breakpoints were associated with open chromatin histone marks, i.e., H3K4Me1, H3K4Me3, and H3K27Ac. We referred to the data sets of seven cell types (GM12878, H1-hESC, HSMM, HUVEC, K562, NHEK, and NHLF) in the UCSC Genome Browser [18]. Fourth, replication timing of breakpoint-containing genomic regions was assessed as described previously [19]. We employed the data of tested loci closest to each breakpoint. Lastly, we analyzed the frequency of de novo SNVs in the 100-bp regions around the breakpoints. To this end, we compared sequence data of the patient and his parents.

### SNVs analyses using the whole genome data

First, to examine whether the patient’s genome was vulnerable to de novo point mutations, we counted the total number of de novo SNVs in the patient. We called de novo SNVs in the entire genome as described previously [20]. Next, to identify disease-associated SNVs in the patient or his father, we searched for protein-altering variants and splice site substitutions in 302 genes known to be involved in genomic stability [21, 22]. Sequence reads were mapped against the human genome reference (hg19/GRCh37) using BWA. We used Picard 2.8.1 to remove duplicate reads and to verify mate-pairs. Local realignment, base quality score recalibration, variant calling, and variant annotation were performed with the Genome Analysis Toolkit 3.5 (Broad Institute) and ANNOVAR [23]. The analyzed genes are shown in Additional file 1: Table S1 [21, 22]. To exclude common polymorphisms, we referred to the data of the 1000 Genomes Project [24], Exome Aggregation Consortium [25], and Human Genetic Variation Database [26]. SNVs that were predicted as benign/tolerated by all of the five in silico programs, namely, Sorting Intolerant From Tolerant [27], PolyPhen-2 HVAR [28], MutationTaster [29], Combined Annotation Dependent Depletion [30], and Mendelian Clinically Applicable Pathogenicity Score [31] were excluded as probably benign polymorphisms. SNVs of interest were confirmed by Sanger sequencing.

## Results

### Identification of a patient with mdnCNVs

**Fig. 1:**
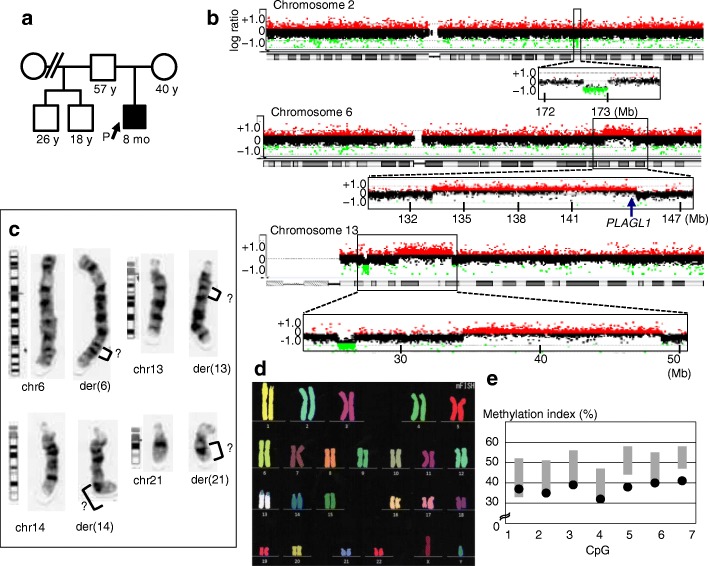
Familial information and cytogenetic findings of the patient. **a** Pedigree of the patient. The proband is indicated by an arrow. All other family members were clinically unremarkable. **b** Array-based comparative genomic hybridization showing large heterozygous copy number variations (CNVs) on chromosomes 2, 6, and 13. Large CNVs were absent from the other chromosomes. The green and red dots denote decreased (log ratio ≤ − 0.8) and increased (log ratio ≥ + 0.4) copy numbers, respectively. Numbers at the bottom of each figure indicate genomic positions based on the Human Genome Database (hg19/build 37). The position of *PLAGL1*, a causative gene for transient neonatal diabetes mellitus, is indicated by the blue arrow. The average log ratios for probes located in deletions and duplications were around − 1.0 and + 0.58, respectively, excluding somatic mosaicism. **c** G-banding of the rearranged chromosomes 6, 13, 14, and 21. No apparent abnormalities were observed on the remaining chromosomes. **d** Multicolor fluorescent in situ hybridization showing the lack of interchromosomal translocations. **e** DNA methylation analysis showing mild hypomethylation of seven CpG sites within *PLAGL1*. The black dots depict methylation indexes of the patient. The gray bars denote reference ranges based on data from 49 unaffected individuals

Genome-wide copy-number analyses for ~ 2100 individuals identified a 1-year and 11-months-old boy with mdnCNVs (Fig. 1a). The patient carried copy number losses on 2q and 13q, and copy number gains on 6q and 13q (Fig. 1b).

### Clinical and cytogenetic analyses of the patient

The patient was born to non-consanguineous Japanese parents. His 57-year-old father, 40-year-old mother, and two half-brothers were clinically unremarkable. The mother had no episodes of abortion. The mother underwent artificial insemination with the husband’s semen, but she received no further assisted reproductive technology. The patient showed intrauterine growth restriction during the third trimester. He was born at 38 weeks and 3 days of gestation with birth weight of 1830 g (− 3.2 SD) and length of 41.8 cm (− 3.0 SD). He repeatedly exhibited pre-feeding hyperglycemic episodes (blood glucose, 17.0–22.4 mmol/L; reference range, 2.8–5.0 mmol/L) from 8 days of age and received insulin injections from 9 days of age. Insulin therapy resulted in a satisfactory control of blood glucose levels and was discontinued at 40 days of age. The patient received a diagnosis of TNDM. In addition, he manifested several congenital anomalies such as cleft palate, iris coloboma, ventricular septal defect, hydronephrosis, umbilical hernia, and clubfoot. He required tracheostomy for laryngomalacia. On the latest visit at 1 year and 11 months of age, the patient showed short stature; his stature and weight were 76.7 cm (− 2.7 SD) and 10.3 kg (− 0.9 SD), respectively. He had no severe developmental delay; he showed head control at 5 months of age, rolling over at 7 months of age, and became able to walk with support at 1 year and 4 months of age.

G-banding analysis for the patient demonstrated a karyotype of 46,XY,der (6) add (6)(q23.3),der (13) add (13)(q12.1),der (14) add (14)(q31),der (21) del(q11.2) add(q11.2) in all 20 cells tested (Fig. 1c and Additional file 2: Figure S1). Multicolor fluorescent in situ hybridization ruled out interchromosomal translocations (Fig. 1d).

### DNA methylation analyses

The patient exhibited mild hypomethylation of the seven CpG sites at 6q24.2 (Fig. 1e), which can be ascribed to copy number gain of the paternally derived (unmethylated) allele. Thus, TNDM of the patient appeared to be associated with 6q duplication on the paternally derived chromosome. The other clinical features of the patient were speculated to result from copy number changes of some genes on the affected chromosomes (Additional file 1: Table S2).

### The origin of the genomic rearrangements

The genomic structure of this case was determined by microsatellite genotyping, short-read paired-end whole-genome sequencing, haplotype-phasing via synthetic long-read whole-genome sequencing, and breakpoint characterization. The average depths of short-read paired-end whole-genome sequencing of the patient and his mother were 39.1 and 38.5, respectively. The percentage of bases with a read depth of more than 20 was 94.8 and 97.0% in the patient and in his mother, respectively. The average depths of synthetic long-read whole-genome sequencing of the patient and his father were 66.6 and 42.4, respectively. The percentage of bases with a read depth of more than 20 was 96.7 and 89.4% in the patient and in his father, respectively.

**Fig. 2:**
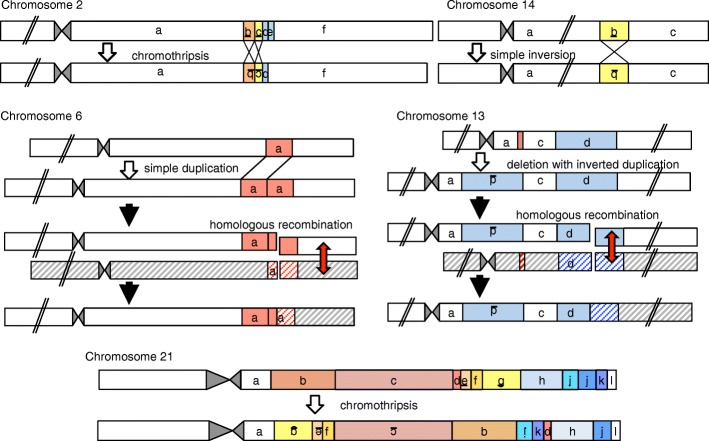
Schematic of predicted mutagenic processes for each rearrangement. Rearranged DNA fragments are highlighted in different colors. Underlined letters depict inverted DNA fragments. Striped boxes indicate non-sister chromatids in the father. Red arrows denote physiological homologous recombination during meiosis. This figure is not drawn to scale

The patient carried a simple tandem duplication on 6q, a simple balanced inversion on 14q, two tandem inversions with a deletion on 2q, an inverted duplication with a deletion on 13q, and catastrophic rearrangements on 21q (Additional file 1: Table S3 and Fig. 2). The profound genomic changes on 2q and 21q were indicative of chromothripsis or chromoanasynthesis [2, 3], although the size of the affected region on 2q was small compared to those of previously reported cases with germline chromothripsis/chromoanasynthesis [5]. Chromothripsis was particularly likely, because the rearrangements on 2q and 21q lacked chromoanasynthesis-specific features, such as copy number gains and templated events [5].

**Fig. 3:**
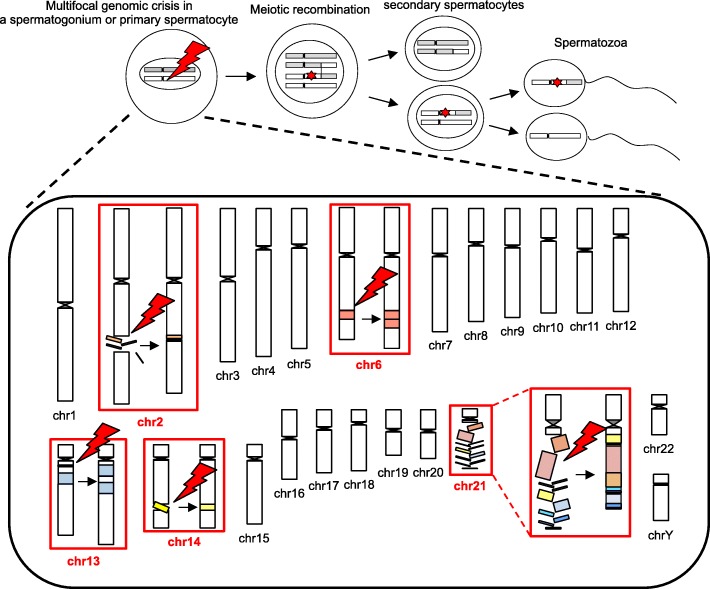
Schematic of multifocal genomic crisis in the patient. Red symbols indicate genomic crisis that have occurred on five chromosomes. Red stars in the upper panel depict chromosomal alterations caused by the genomic crisis

All genomic changes in the boy resided on paternally derived chromosomes; there was no data to suggest the involvement of maternal material in the rearrangements (Table 1 and Additional file 1: Table S4 and S5). The de novo occurrence of the rearrangements was confirmed by the negative results of PCR-amplification for all 20 fusion junctions from the parental DNA samples (Additional file 2: Figure S2). The duplicated regions of chromosomes 6 and 13 entailed both triallelic and diallelic genotypes (Table 1 and Additional file 1: Table S4), and all SNVs flanking each breakpoint seemed likely to belong to the same haplotype of the father (Additional file 1: Table S5). These results indicate that the rearrangements on 6q and 13q initially occurred during premeiotic mitosis in a testicular germ cell, and were subsequently modified through physiological homologous recombination during meiosis I (Figs. 2 and 3).

**Table 1:** Results of microsatellite analysis

Locus	Chromosomal position^a^	Patient^b^	Father^b^	Mother^b^	Assessment
D6S270	chr6:134654832–134654974 (6q23.2)	139	139/141	129/139	diallelic
D6S292	chr6:136315223–136315379 (6q23.3)	145/145/157	145/157	145/151	triallelic
D6S1569	chr6:139053750–139053879 (6q24.1)	129/133/135	129/135	133	triallelic
D6S308	chr6:141256724–141256922 (6q24.1)	194/198/198	194/198	198/200	triallelic
D13S218	chr13:39032331–39032519 (13q13.3)	188/190/192	188/192	190	triallelic
D13S263	chr13:42080976–42081126 (13q14.11)	141/147/149	141/147	147/149	triallelic

^a^Genomic positions correspond to the human genome reference assembly (UCSC Genome Browser, hg19/GRCh37)

^b^The numbers indicate PCR product size (bp)

### Genetic and genomic backgrounds of the rearrangements

We further investigated genetic and genomic features of the patient (Additional file 1: Table S6 and Additional file 2: Figure S3). Most breakpoints resided outside of repetitive sequences. Microhomologies or microhomeologies at the fusion junction were observed on 6q and 13q, but not on 2q, 14q, or 21q. One fusion junction on 21q was accompanied by an untemplated 7-bp insertion. The results for 2q and 21q were consistent with chromothripsis [5]. The rearrangements on 13q were indicative of replication-based errors; however, we cannot exclude the possibility that these rearrangements also resulted from chromothripsis, because duplications and microhomology have been described in some cases of chromothripsis [32–34]. Simple rearrangements on 6q and 14q likely resulted from NHEJ or replication-based errors.

The breakpoint-flanking regions were neither enriched with de novo SNVs nor associated with a specific replication timing or histone marks. The total number of de novo SNVs in the patient’s genome was only slightly higher than the average of people with the same parental age (152 vs. 112) [20]. The sole protein-altering de novo SNV was p.R2768H in *DNAH2*, a gene encoding the axonemal dynein heavy chain. Exome sequencing of the patient and his father identified no pathogenic mutations in 302 genes involved in genomic stability, except for a heterozygous p.A419T substitution in *RAD50*, a gene involved in the maintenance of chromosomal stability [21, 22], in the father. Repeated aCGH using patient’s DNA obtained from peripheral blood and oral swab at 2-weeks and at 8-months of age yielded identical results, excluding the possibility of postnatal genomic instability. Moreover, PCR analysis invariably amplified DNA fragments containing the fusion junctions from the patient’s peripheral blood, oral swab, and hair follicle cells, suggesting the lack of somatic mosaicism.

## Discussion

We identified a boy who carried five clusters of large de novo rearrangements, all of which were generated during spermatogenesis. Considering the rarity of multiple de novo rearrangements on human chromosomes [35], genomic lesions in this case are more likely to reflect a specific mutagenic event than the accumulation of five independent abnormalities. Our data indicate that the spermatogenic process includes a brief window of time during which multifocal genomic crisis can develop, although it remains unknown whether all rearrangements in the present case share a common origin or result from different cellular events induced by a single trigger. Notably, the present case shares several common features with the previously reported mdnCNV cases, as follows: (i) multiple rearrangements were created exclusively in perizygotic cells, (ii) the rearrangements were non-recurrent and affected much larger regions than typical human CNVs, (iii) each rearrangement cluster was restricted to one chromosomal arm and did not involve interchromosomal translocations, and (iv) the breakpoints were often associated with microhomology and microhomeology [5, 7]. Our results support the hypothesis of Liu et al. that there is a specific window in early human development permissive to large chromosomal rearrangements due to NHEJ or replication-based errors [7]. Unlike de novo SNVs that are known to occur predominantly in late-replicating regions [36], mdnCNVs appear to originate independently of replication timing. Moreover, since the rearrangements in our case and the other mdnCNV cases reside at various positions in the genome and are not associated with repeat sequences or open chromatin histone marks, local genomic environments are unlikely to play critical roles in the development of such rearrangements.

More importantly, the present case exhibited several unique characteristics. First, this case encompassed various types of rearrangements, including simple duplications, simple inversions, and chromothriptic changes. These results challenge the current understanding of the etiology of de novo genomic abnormalities. Thus far, chromothripsis is predicted to result from micronucleus-mediated reconstruction of mis-segregated or dicentric chromosomes, while simple CNVs are ascribed to NAHR, NHEJ, or replication-based errors [1, 32–34]. The results of the present case can be explained by assuming that several micronuclei can concurrently develop in a testicular germ cell and create both simple and catastrophic rearrangements. Alternatively, a hitherto unrecognized micronucleus-independent mechanism may produce multifocal intrachromosomal reconstructions, including chromothripsis. Indeed, the spatially confined genomic chaos of 2q may result from atypical chromothripsis. Second, the genomic crisis in this case appears to have occurred during premeiotic mitosis. Specifically, the rearrangements likely originated in a spermatogonium or primary spermatocyte. This is in contrast to previous mdnCNV cases, in which most rearrangements were created in primary oocytes and postzygotic cells [7]. Moreover, none of the breakpoints in our case were accompanied by de novo SNVs, which were significantly enriched in the breakpoint-flanking regions of mdnCNVs [7]. In addition, the mutagenic event in our case did not particularly favor tandem duplications. These findings suggest etiological and consequential differences between our case and the other mdnCNV cases. Thus, the present study significantly broadens the concept of mdnCNVs. In this regard, germline chromothripsis was shown to occur during spermatogenesis, but not during oogenesis [1, 6]. It is possible that the multifocal genomic crisis in our case leading to chromothripsis was driven by some spermatogenesis-specific factors. Lastly, the present patient was born to a 57-year-old father. Advanced paternal age may have contributed to the development of rearrangements, although there are no data regarding the parental ages of the previous mdnCNV cases. Indeed, chromosomal damage may gradually accumulate in spermatogonia [37]. Moreover, aging is known to accelerate telomere dysfunction [38], which could trigger chromothripsis [6, 34]. However, since previous studies have shown that paternal aging has relatively minor effects on the frequency of de novo CNVs [39, 40], the extreme genomic changes in this case cannot be explained by advanced paternal age alone. In addition, while the father carried a rare SNV in *RAD50* and the patient had a de novo SNV in *DNAH2*, the pathogenicity of these variants remains unknown.

## Conclusions

In summary, the results indicate that a transient multifocal genomic crisis in prezygotic germ cells can introduce several chromothriptic and non-chromothriptic changes into the genome. The genomic structure of the present case showed not only some similarities but also striking differences to those of previously reported mdnCNV cases, expanding the concept of the “organismal CNV mutator phenotype”. This study provides insights into the cellular events that alter the global chromosomal architecture of human embryos.

## Additional files

Additional file 1: **Table S1.** List of genes analyzed for pathogenic SNVs. **Table S2.** Genes and transcripts affected by the chromosomal rearrangements. **Table S3.** Summary of de novo rearrangements in previously reported cases and the present case. **Table S4.** Representative results of SNV-genotyping of the duplicated regions on chromosomes 6 and 13. **Table S5.** Representative results of haplotype-phasing. **Table S6.** Characteristics of the breakpoints. (XLSX 386 kb)

Additional file 2: **Figure S1.** G-banding of the patient. **Figure S2.** Representative results of the PCR-amplification of DNA fragments containing the fusion junctions. **Figure S3.** Breakpoint structures of the rearrangements. (PDF 140 kb)

## Abbreviations

aCGH: Array-based comparative genomic hybridization
BWA: Burrows-Wheeler Aligner
CNV: Copy number variant
HERV: Human endogenous retrovirus
IGV: Integrative Genomic Viewer
LINE: Long interspersed nuclear element
mdnCNV: Multiple de novo copy number variant
NAHR: Non-allelic homologous recombination
NHEJ: Non-homologous end-joining
SINE: Short interspersed nuclear element
SNV: Single nucleotide variant
TNDM: Transient neonatal diabetes mellitus
