# Supplementary material for: Transient multifocal genomic crisis creating chromothriptic and non-chromothriptic rearrangements in prezygotic testicular germ cells
Source: BMC Med Genomics. 2019 May 28;12:77. doi: 10.1186/s12920-019-0526-3 (PMC6540402; doi:10.1186/s12920-019-0526-3)
Supplement: Supplementary file 2 — Figure S1. G-banding of the patient. Figure S2. Representative results of the PCR-amplification of DNA fragments containing the fusion junctions. Figure S3. Breakpoint structures of the rearrangements. (PDF 140 kb) [file 12920_2019_526_MOESM2_ESM.pdf]

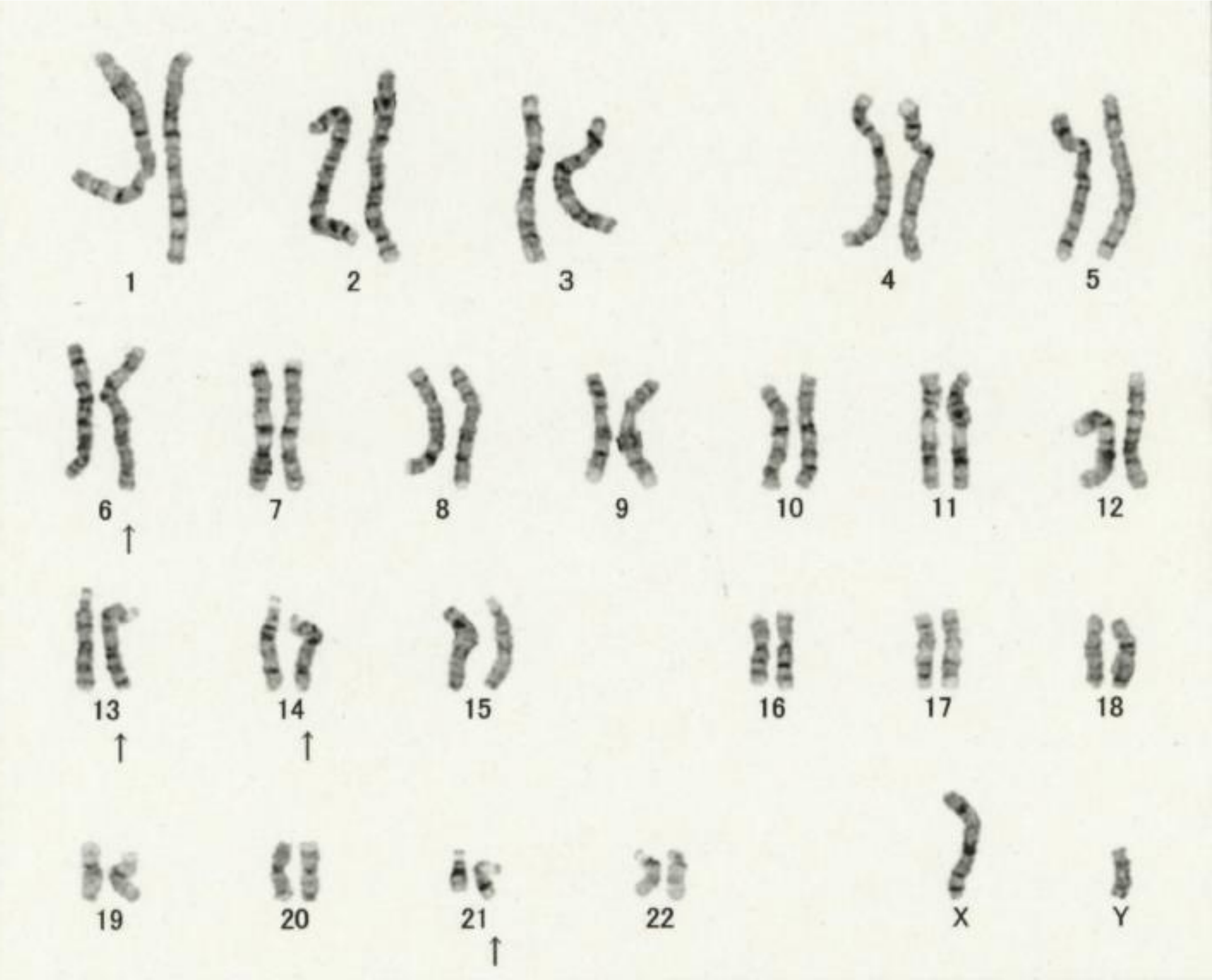

Additional file 2: Fig. S1 G-banding of the present patient. Arrows point chromosomes with aberrant structure.

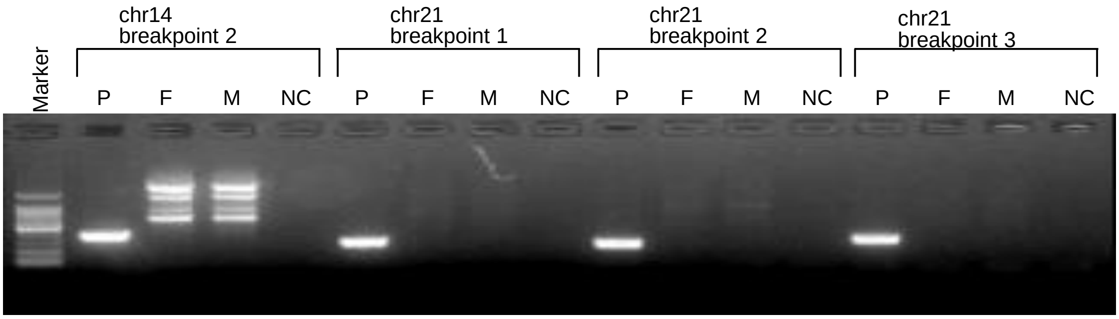

Additional file 2: Fig. S2 Representative results of the PCR-amplification of DNA fragments containing the fusion junctions. The numbers of the breakpoints correspond to those of Fig. S3. P, F, M, and NC denote the patient, father, mother, and negative control, respectively.

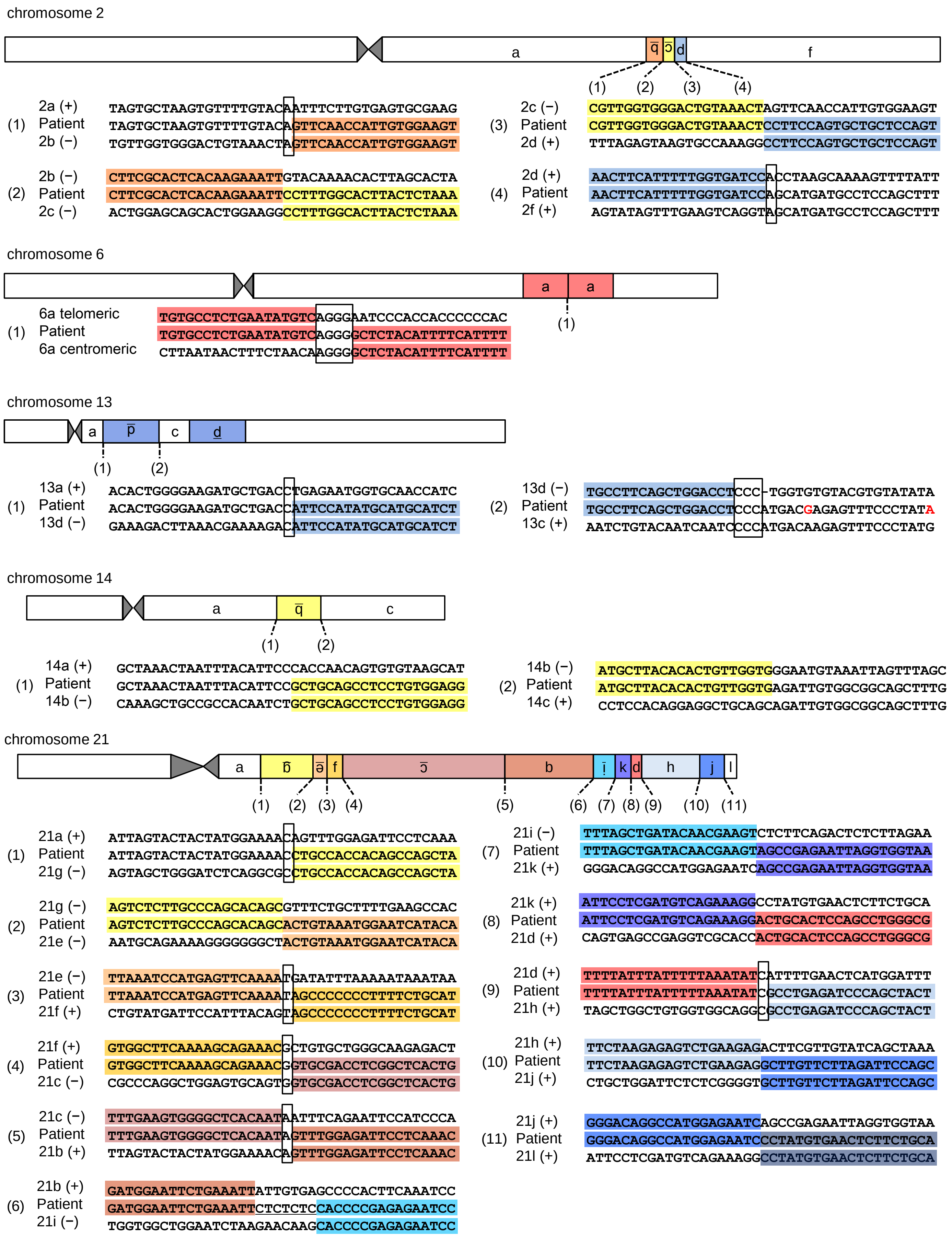

Additional file 2: Fig. S3 Breakpoint structures of the rearrangements. Sequences of the patient are aligned with the human reference data (GRCh37 on Ensemble Genome Browser 91, http://grch37.ensembl.org/Homo_sapiens/Info/Index). Microhomologies at the fusion junctions are shown in boxes. An untemplated insertion is underlined. The (+) and (−) symbols indicate forward (CEN → q-ter) and reverse (q-ter → CEN) directions, respectively. Red letters on chromosome 13 denote single nucleotide variants shared by the patient and his father.
